# A Novel *LMX1A* Frameshift Variant Underlies Familial Phenotypic Heterogeneity in DFNA7

**DOI:** 10.1155/humu/9930672

**Published:** 2026-05-30

**Authors:** Chenyang Xu, Zhipeng Nie, Suyang Wang, Yiming Zhu, Xiaowen Liu, Yufen Guo

**Affiliations:** ^1^ The Second Clinical Medical School, Lanzhou University, Lanzhou, Gansu, China, lzu.edu.cn; ^2^ Department of Otolaryngology-Head and Neck Surgery, Lanzhou University Second Hospital, Lanzhou, Gansu, China, ldey.cn; ^3^ Center for Mitochondrial Biomedicine and Department of Ophthalmology, Zhejiang University School of Medicine, Zhejiang, China, zju.edu.cn; ^4^ Department of Otolaryngology-Head and Neck Surgery, Maternal and Child Health Hospital of Gansu Province, Lanzhou, Gansu, China; ^5^ Department of Otolaryngology-Head and Neck Surgery, Gansu Provincial Hospital, Lanzhou, Gansu, China, gsyy.cn

**Keywords:** asymmetric sensorineural hearing loss, auditory homeostasis, cochlear aperture stenosis, *LMX1A*, transcriptional activity

## Abstract

Pathogenic variants in the LIM‐homeodomain transcription factor *LMX1A* represent a rare yet critical etiology for autosomal dominant nonsyndromic hearing loss 7 (DFNA7) and less frequently, its autosomal recessive counterpart (ARNSHL). Here, we describe a novel heterozygous frameshift variant, *LMX1A* c.405delT (p.Phe135LeufsTer3), identified in a three‐generation Chinese family, cosegregating with progressive and asymmetric sensorineural hearing loss (ASNHL). Clinical manifestations exhibited significant intrafamilial phenotypic variability, with hearing loss (HL) severity ranging from mild to profound, and onset varying from infancy to mid‐adulthood. High‐resolution imaging revealed bilateral cochlear aperture stenosis (CAS) in the severely affected proband. Whole‐exome sequencing (WES) and cosegregation analysis confirmed this novel variant. Structural modeling predicted the truncation of both the DNA‐binding homeodomain and the C‐terminus. Subsequent reporter assays demonstrated a significant loss of transcriptional activity. Furthermore, plasmid titration experiments and Actinomycin D chase assays functionally corroborated the haploinsufficiency mechanism and excluded the dominant‐negative effect. Integrative multiomics profiling (RNA‐seq and DIA‐based proteomics) of in vitro HEI‐OC1 model revealed molecular perturbations following *Lmx1a* deficiency, primarily involved in synaptic signaling and immune‐inflammatory cascades. This study broadens the *LMX1A* mutational landscape, refines the clinical phenotypic spectrum of DFNA7 and establishes insufficient *LMX1A* dosage as the primary disease driver.

## 1. Introduction

Hereditary hearing loss (HHL) is a genetically driven disorder that profoundly impairs communication and quality of life. It shows marked genetic and allelic heterogeneity; mutations in different genes can converge on similar phenotypes, whereas distinct variants within the same gene may yield divergent clinical presentations. HHL is broadly divided into syndromic hearing loss (SHL), which presents alongside additional systemic features, and non‐syndromic hearing loss (NSHL), wherein auditory impairment represents the sole clinical manifestation. Constituting approximately 70% of all HHL cases, NSHL exhibits a wide array of inheritance patterns, encompassing autosomal recessive (AR) (DFNB), autosomal dominant (AD) (DFNA), X‐linked, mitochondrial, and Y‐linked traits [1]. Although DFNA is less prevalent than DFNB, its familial burden remains substantial, driven by vertical transmission and a 50% recurrence risk per generation. Clinically, it predominantly manifests as progressive, postlingual sensorineural hearing loss (HL).

**Figure 1:**
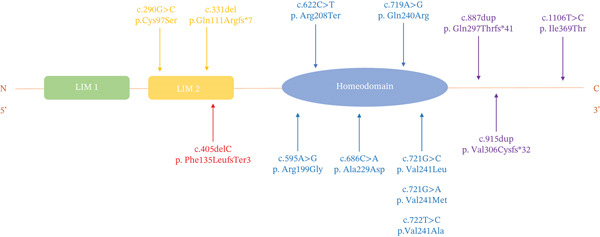
Schematic of LMX1A domains (LIM1, LIM2, homeodomain, C‐terminus) with locations of known pathogenic variants and the novel c.405delT (p.Phe135LeufsTer3) reported here. Red arrow: variant identified in this study.

Specifically, the DFNA7 locus is tightly linked to pathogenic variants within the *LMX1A* gene. Clinical features associated with DFNA7 exhibit considerable expressivity, including highly variable age of onset (ranging from congenital to mid‐adulthood), severity, progression rate, and expression among affected family members. To date, only 12 unique pathogenic *LMX1A* variants have been reported to be associated with DFNA7, including a homozygous missense mutation observed in a family linked to an atypical autosomal recessive non‐syndromic hearing loss (ARNSHL) [2–9] (Figure 1, Table 1).

**Table 1:** *LMX1A* variants in the previous study and their audiological phenotype associated with DFNA7. (N/A, not available; VUS, variant of uncertain significance; Het, heterozygous; Homo, homozygous).

Nation	Zygosity	Inheritance	Mutation	Mutation Type	Exon	Domain	Patient	Sex and Age	Onset	Audiologic	Hearing threshold (Left)	Hearing threshold (right)	Audioprofile	Laterality	HL progression	Vestibular dysfuction	ACMG Criteria	Pathogenic
Dutch [8]	Het	Dominant	c.721G > C p.Val241Leu	Missense	6	Homeodomain	II‐7	F/56 (First evaluation)	27	PTA	35‐75‐80‐80‐na	40‐80‐80‐90‐105	Downsloping	No	N/A	No	PM2, PP3, PP1, PS3	Pathogenic
								F/73 (Second evalution)			55‐65‐75‐65‐na	60‐80‐80‐90‐100						
							III‐8	F/24 (First evaluation)	Puberty	PTA	35‐30‐20‐55‐55	25‐40‐45‐50‐55		No	Yes	Yes		
								F/42 (Second evalution)			40‐60‐60‐55‐65	40‐60‐70‐70‐80						
							IV‐2	F/6 (First evaluation)	Congenital	PTA	10‐15‐30‐40‐60	45‐55‐60‐55‐70		Asymmetric	No	No		
								F/9 (Second evalution)			20‐15‐35‐70‐90	40‐50‐55‐55‐65						
	Het	Dominant	c.290G > C p.Cys97Ser	Missense	4	LIM2	I‐2	F/85	Childhood	PTA	80‐90‐80‐80‐100	80‐75‐85‐85‐95	Downsloping	No	Yes	No	PM2, PP3, PS3, PP1	Likely pathogenic
							II‐2	F/26 (First evaluation)	26	PTA	10‐5‐0‐15‐10	35‐15‐20‐45‐50		Asymmetric	Yes	Yes		
								F/54 (Second evaluation)			25‐30‐30‐45‐55	50‐60‐65‐65‐75						
							II‐3	M/15	Congenitial	PTA	80‐90‐90‐80‐80	80‐80‐80‐80‐80		No	Yes	Yes		
								M/52 (Second evalution)			100‐120‐120‐120‐110	100‐115‐120‐120‐110						
							II‐4	M/30 (First evaluation)	35	PTA	35‐30‐30‐35‐50	20‐20‐40‐40‐40		No	Yes	Yes		
								M/40 (Second evalution)			55‐55‐60‐50‐75	40‐45‐50‐60‐50						

Pakistan [7]	Homo	Recessive	c.1106T>C p.Ile369Thr	Missense	9	C‐terminus	IV‐1	M/15	Congenital	PTA	85‐75‐90‐95‐95	90‐85‐95‐95‐100	Flat	No	No	No	PM2, PP3, PP1	Pathogenic
							IV‐2	M/12	Congenital	PTA	85‐85‐90‐90‐95	90‐85‐95‐100‐100		No		No		
Korea [5]	Het	Dominant (de novo)	c.595A > G p.Arg199Gly	Missense	5	Homeodomain (N‐terminal arm)	927	M/3m	Congenital	ASSR/ABR	90‐90‐70‐70	100‐100‐100‐110	Flat	Asymmetric	Yes	N/A	PM2,PP3,PS2,PS3	Likely pathogenic
Poland [6]	Het	Dominant	c.686C>A p.Ala229Asp	Missense	6	Homeodomain	II‐5	F/70	40	PTA	60‐65‐75‐85‐120	60‐65‐75‐85‐120	Downsloping	N/A	N/A	No	PM2,PP3,PP1,PS3	VUS
							III‐2	F/40	30	PTA	25‐30‐40‐55‐50	25‐30‐40‐55‐50		N/A	N/A	No		
							IV‐1	F/8	Childhood(<7)	PTA	10‐15‐45‐45‐55	10‐15‐45‐45‐55		N/A	Yes	No		

China [9]	Het	Dominant	c.915dup p.Val306Cysfs∗32	Frameshift	8	c‐terminus	II‐1	M/60	Middle age	PTA	45‐50‐50‐75‐90	45‐50‐50‐95‐90	Downsloping	No	N/A	No	PVS1,PM2,PP1	Pathogenic
							II‐3	M/58	30	PTA	85‐90‐80‐80‐70	90‐95‐90‐95‐70	Flat	No	N/A	No		
							II‐6	M/52	Middle age	PTA	25‐20‐35‐60‐65	60‐25‐40‐95‐100	Downsloping	Asymmetric	N/A	No		
							II‐9	F/47	Congenital	PTA	115‐120‐120‐115‐105	115‐120‐120‐115‐105	Flat	No	Yes	No		
							III‐7	F/26	Congenital	PTA	85‐90‐80‐80‐70	90‐95‐90‐95‐70	Flat	No	Yes	No		
							III‐8	M/28	N/A	PTA	20‐20‐20‐25‐40	15‐15‐20‐25‐35	Downsloping	No	Yes	No		

Korea [5]	Het	Dominant	c.622C>T p.Arg208∗	Nonsense	5	Homeodomain	SB727‐1294	F/31	Early 10s	PTA	75‐80‐80‐100‐110	35‐50‐50‐60‐65	Downsloping	Asymmetric	Yes	No	PVS1, PM2, PP1	Pathogenic
	Het	Dominant	c.719A>G p.Gln240Arg	Missense	6	Homeodomain	SB742‐1317	F/2m	Congenital	ASSR/ABR	100‐100‐100‐100	45‐45‐45‐45	Flat	Asymmetric	N/A	N/A	PM2, PP3, PP1	VUS
							SB742‐1657	F/60	Early 20s	PTA	40‐65‐60‐65‐80	50‐60‐70‐65‐80	Downsloping	No	Yes	No		
	Het	Dominant	c.721G>A p.Val241Met	Missense	6	Homeodomain	SH421‐906	F/2m	Late 10s	ASSR	50‐70‐60‐50	50‐40‐40‐40	Flat	Asymmetric	Yes	N/A	PM1, PM2, PM5, PS3, PP3	Likely pathogenic
							SH421‐907	M/24 (Before use hearing aids)	Late 10s	PTA	40‐40‐45‐50‐NA	110‐110‐110‐110‐NA	Downsloping	Asymmetric	Yes	No		
								M/31 (Use hearing aids 7 years)			25‐40‐50‐75‐NA	95‐115‐115‐120‐NA						
	Het	Dominant	c.887dup p.Gln297Thrfs∗41	Frameshift	8	C‐terminus	SH407‐878	M/21	Early 20s	PTA	50‐55‐35‐30‐NA	55‐70‐60‐55‐NA	Downsloping	Asymmetric	N/A	Yes	PVS1, PM2, PS2	Pathogenic

Korea [3]	Het	Dominant	c.331del p.Gln111Argfs∗7	Frameshift	4	LIM2	SH512‐1	N/A	3 months	N/A	Moderate		N/A	No	N/A	N/A	PVS1, PM2	Likely pathogenic
							SH512‐2	N/A	1 year	N/A	Severe		Downsloping	No				
European descent [2]	Het	Dominant	c.722T>C p.Val241Ala	Missense	6	Homeodomain	IV:3	M/56	Adolescence	PTA	85‐95‐105‐110‐105‐115‐115‐100‐95	90‐105‐110‐110‐110‐115‐115‐100‐95	Flat	No	Yes	No	PM1, PM2, PM5, PP3	Likely pathogenic
							V:5	M/12	Congenital	N/A	N/A		N/A	N/A	N/A			

China (This study)	Het	Dominant	c.405delT p.Phe135LeufsTer3	Frameshift	4	LIM2	I‐2	M/52	Early 30s	PTA	30‐50‐70‐75‐NA	35‐45‐75‐70‐85	Downsloping	No	Yes	Yes	PVS1, PS3, PM2, PP1	Pathogenic
							II‐1	F/16	Congenital	PTA	85‐80‐75‐65‐75	80‐75‐75‐65‐65	Flat	No	Yes	Yes		
							II‐2	M/28	Unkonwn	PTA	20‐15‐10‐30‐40	10‐10‐5‐15‐40	Downsloping	No	Yes	No		
							III‐1	F/2	Congenital	ASSR/ABR	85‐80‐80‐70	100‐100‐100‐100	Flat	Asymmetric	N/A	No		

In this study, we identified a novel heterozygous frameshift variant, *LMX1A* c.405delT (p.Phe135LeufsTer3, RefSeq: NM_177398), which segregates with autosomal dominant non‐syndromic hearing loss (ADNSHL) in a three‐generation Chinese family. The pedigree displays marked variable expressivity (HL thresholds ranging from mild to profound, and onset ages from infancy to adulthood). Notably, the proband presented with bilateral cochlear aperture stenosis (CAS), a structural anomaly that potentially expands the phenotypic spectrum of *LMX1A* variants and merits clinical attention. By functionally characterizing this mutation and exploring the downstream molecular landscape, this study is aimed at further characterizing the genotype–phenotype correlations of DFNA7 and elucidate the pathogenic mechanisms underlying *LMX1A*‐related HL.

## 2. Materials and Methods

This study was approved by the Ethics Committee of the Lanzhou University Second Hospital. All procedures conformed to the Declaration of Helsinki. Written informed consent was obtained from all participants or legal guardians. This study involved only human peripheral blood samples; no solid tissue specimens were collected, and no live animal experiments were performed. All methods were executed in accordance with the relevant guidelines and regulations.

The cell lines used in this study, HEK293 (RRID: CVCL_0045) and HEI‐OC1 cells (RRID: CVCL_D899), were, respectively, obtained from Dr. Suyang Wang and Qingke Biotechnology Company. All cell lines have been routinely tested for mycoplasma contamination using the Myco‐LumiTM Luminescent Mycoplasma Detection Kit (C0297, Beyotime, Shanghai, China).

### 2.1. Families, Clinical Evaluation and Peripheral Blood Sample Collection

A three‐generation family with NSHL presented to the Department of Otolaryngology‐Head and Neck Surgery at Lanzhou University Second Hospital. A comprehensive medical history was obtained and potential secondary causes of HL, such as infections, ototoxic drug exposure, and trauma, were thoroughly evaluated and excluded. A general physical examination was also performed to rule out any other systemic conditions.

Otological examination included inspection of the external ear and otoscopy to assess the tympanic membrane integrity and middle ear aeration. Comprehensive audiological assessments included pure‐tone audiometry (PTA), tympanometry, acoustic reflex testing, auditory brainstem responses (ABR), auditory steady‐state response (ASSR), and speech audiometry. PTA was conducted in accordance with established clinical standards. Air conduction (AC) thresholds were measured at 0.25, 0.5, 1.0, 2.0, 4.0, and 8.0 kHz in dB HL, and bone conduction (BC) thresholds were evaluated at 0.5, 1.0, 2.0, and 4.0 kHz in dB HL. The pure‐tone average was calculated at 0.5, 1, 2, and 4 kHz to assess the severity and exclude any conductive components. HL severity was categorized as subtle (16–25 dB HL), mild (26–40 dB HL), moderate (41–70 dB HL), severe (71–95 dB HL), or profound (> 95 dB HL) based on World Health Organization criteria [10]. Audiogram configurations were classified as upsloping, downsloping, U‐shaped (cookie bite), or flat. ASNHL was defined as an interaural asymmetry of ≥ 20 dB HL at two contiguous frequencies or ≥ 15 dB HL at any two frequencies between 2000 and 8000 Hz [11]. High‐resolution computed tomography (HRCT) and magnetic resonance imaging (MRI) of the temporal bones were performed on the proband (III: 1) to evaluate cochlear and vestibular malformations. In cases of discrepant interpretations, two independent otologists reviewed the images to reach a consensus, specifically assessing the presence of enlarged vestibular aqueduct (EVA) and CAS [12–14].

Peripheral blood samples (2 mL) were obtained from all six individuals (I: 1, I: 2, II: 1, II: 2, II: 3, and III: 1) in the family, as well as from 500 healthy controls and 122 unrelated Chinese families with HL.

### 2.2. Whole‐Exome Sequencing (WES) and Variant Analysis

WES was performed on individuals II: 2, II: 3, and III: 1. Genomic DNA was isolated from the peripheral blood via phenol‐chloroform extraction and quantified using NanoDrop 2000 spectrophotometer (Thermo Fisher Scientific, Waltham, Massachusetts, United States). Mutations in GJB2, SLC26A4, and mitochondrial 12 s rRNA genes were excluded using Sanger sequencing. Following shearing, DNA fragments were ligated into Illumina‐compatible adapters. The resulting libraries were then captured using a GenCap Capture Kit (MyGenostics, Beijing, China, Probe Set: CM1160). Subsequently, the enriched libraries were sequenced on an Illumina NovaSeq 500 platform (Illumina Inc., San Diego, California, United States) using 2 × 150 bp paired‐end sequencing, achieving an average output of 80–100 million reads/sample. Subsequently, raw reads were aligned to the human reference genome (GRCh37/hg19) using the Burrows–Wheeler Aligner (BWA) [15]. The resulting alignments were sorted using SAMtools v1.2[16] and filtered using BamTools [17] v2.4.0. Single nucleotide polymorphisms (SNPs) and small insertions/deletions variants were called using the Genome Analysis Toolkit (GATK) [18] v4.0.8.1. Variant annotation, including functional predictions and allele frequency filtering based on population databases (Genome Aggregation Database [gnomAD], 1000 Genomes), was performed using ANNOVAR [19].

### 2.3. Sanger Validation and Segregation

Sanger sequencing was used to verify the putative causative variants identified within the candidate genes. Additionally, cosegregation analysis was conducted by genotyping all available family members (I: 1, I: 2, and II: 1) via Sanger sequencing to confirm the segregation pattern of the identified variant. Following genomic DNA extraction, target regions were PCR‐amplified utilizing gene‐specific primers designed via SnapGene software (primer sequences are detailed in Table S1). The resulting amplicons were purified using magnetic beads (at a 1.5:1 bead‐to‐sample ratio) and subjected to cycle sequencing with the BigDye Terminator v3.1 (Applied Biosystems, United States). Thermocycling conditions comprised 28 cycles of denaturation at 96°C for 15 s, annealing at 50°C for 6 s, and extension at 60°C for 3.5 min. Postsequencing purification was achieved via ethanol/sodium acetate precipitation (25:1 ratio), followed by capillary electrophoresis on an ABI 3130xl Genetic Analyzer utilizing POP‐7 polymer. The generated sequence chromatograms were visually inspected and aligned against the wild‐type (WT) *LMX1A* sequence (MIM: 600298; RefSeq: NM_177398.4).

### 2.4. Protein Modeling and Multiple Sequence Alignment (MSA)

The amino acid sequence of human *LMX1A* (RefSeq: NP_796372.1) was obtained from the National Center for Biotechnology Information (NCBI) database (https://www.ncbi.nlm.nih.gov).Three‐dimensionalprotein structures for both WT LMX1A and mutant (MT) LMX1A p.Phe135LeufsTer3 were generated using SWISS‐MODEL. Structural visualization and comparative analysis between the WT and MT models were performed using PyMOL. To evaluate the evolutionary conservation of the altered residue, MSA of LMX1A orthologs across diverse species were generated utilizing the HomoloGene database.

### 2.5. Expression Plasmids and Luciferase Assays

A 613‐bp fragment of the human *INS* gene promoter was PCR‐amplified and subcloned into the pGL4.12[luc2CP] vector upstream of the firefly luciferase reporter gene, generating the *INS*‐promoter reporter construct (pGL4.12‐*INS*‐luc) (Table S2). The unmodified pGL4.12‐Luc vector was used as the control plasmid. Site‐directed mutagenesis was performed on a pcDNA3.1 vector harboring the WT *LMX1A* cDNA template to generate six mutant constructs, representing variants across distinct functional domains: c.290G > C (p.Cys97Ser, LIM2 domain), c.405delT (p.Phe135LeufsTer3, LIM2 domain), c.595A > G (p.Arg199Gly, homeodomain), c.721G > C (p.Val241Leu, homeodomain), c.887dup (p.Gln297ThrfsTer41, Homeodomain), and c.1106 T > C (p.Ile369Thr, C‐terminus). HEK293 cells were seeded into 96‐well plates at a density of 2 × 104 cells/well and cultured in DMEM (Gibco) supplemented with 10% FBS at 37°C in a humidified 5% CO2 atmosphere until 70%–80% confluency was achieved. Cells were cotransfected using the Hieff Trans Liposomal Reagent (Yeasen, Cat 40802ES01). Each well received 0.1 *μ*g pGL4.12‐*INS*‐luc, 0.1 *μ*g either WT or mutant LMX1A expression plasmid (pcDNA3.1‐*LMX1A*), and 0.015 *μ*g Renilla luciferase control plasmid (pRL‐TK) for normalization. For each transfection, plasmid DNA was diluted in 20 *μ*L serum‐free DMEM, mixed with 0.3 *μ*L Hieff Trans reagent (1: 3 lipid: DNA ratio), and incubated at room temperature for 25 min to allow complex formation. After 6 h of incubation with transfection complexes, the medium was aspirated and replaced with fresh complete DMEM. Luciferase activity was measured using the Dual‐Luciferase Reporter Assay System, according to the manufacturer′s protocol. The firefly luciferase activity values were normalized to the corresponding Renilla luciferase activity values for each individual well. All reporter assays were independently replicated a minimum of three times. Relative transcriptional activity, expressed as the fold‐change in the normalized Firefly/Renilla luminescence ratio compared with cells cotransfected with the WT LMX1A plasmid and the *INS*‐reporter, was statistically compared between each mutant and WT LMX1A control.

### 2.6. Total RNA Extraction and RNA‐Seq Analysis

HEI‐OC1 cells were transfected with either the vector harboring the WT *Lmx1a* sequence or the vector containing the c.405delT mutant *Lmx1a* sequence. Transfection efficiency was assessed using reverse transcription quantitative PCR (RT‐qPCR) to quantify exogenous *Lmx1a* overexpression (primer sequences are provided in Table S3). Total RNA was extracted from the transfected HEI‐OC1 cells using TRIzol Reagent (Invitrogen, Thermo Fisher Scientific, United States) in accordance with the manufacturer′s protocol. RNA concentration and purity were spectrophotometrically assessed using a Nanodrop 2000 spectrophotometer (Thermo Fisher Scientific). Acceptable samples exhibited A260/A280 ratios of 1.8–2.2, concentrations ≥ 40 ng/*μ*L, and a total yield ≥ 2 *μ*g. RNA integrity was further evaluated using Agilent 2100 Bioanalyzer (Agilent Technologies), which requires an RNA Integrity Number (RIN) ≥ 7.

Strand‐specific RNA sequencing libraries were constructed utilizing the VAHTS Universal V6 RNA‐Seq Library Prep Kit for Illumina according to the manufacturer’s instructions. Briefly, poly (A) + mRNA was enriched using oligo (dT) magnetic beads, followed by double‐stranded cDNA synthesis. Following end‐repair and A‐tailing, Illumina‐compatible adapters were ligated to the cDNA. The adapter‐ligated fragments were then purified using VAHTS DNA Clean Beads and subjected to PCR amplification for enrichment. Final library quality and concentration were assessed using a Qsep400 bio‐fragment analyzer and a Qubit 3.0 fluorometer. Sequencing was executed on an Illumina NovaSeq X Plus platform (Illumina, San Diego, California, United States). Raw sequencing reads were subjected to quality control using FastQC, and the resulting high‐quality clean reads were aligned to the Mus musculus reference genome (GRCm39.112) using HISAT2.

### 2.7. DIA‐Based Quantitative Proteomics Analysis

HEI‐OC1 cells (expressing either WT or mutant LMX1A) were lysed in an 8 M urea buffer containing 1 mM PMSF and 2 mM EDTA. Protein concentrations were determined via a standard BCA assay. For each sample, 100 *μ*g of protein was reduced with 5 mM DTT (37°C, 45 min) and subsequently alkylated with 11 mM iodoacetamide (RT, 15 min, in the dark). Following dilution with 25 mM NH_4_HCO_3_ to reduce urea concentration, samples were digested overnight with Trypsin (Promega) at 37°C. The resulting peptides were acidified to pH 2–3 with trifluoroacetic acid (TFA), desalted using C18 resin (Millipore), and spectrophotometrically quantified. Subsequently, approximately 200 ng of peptides was analyzed using a Vanquish Neo UHPLC system coupled to an Orbitrap Astral mass spectrometer (Thermo Fisher Scientific). Chromatographic separation was achieved on an Easy‐Spray PepMap Neo column (150 *μ*m × 15 cm, 2 *μ*m) maintained at 55°C over an 8‐min total runtime. The mass spectrometer was operated in data‐independent acquisition (DIA) mode spanning a mass range of 380–980 m/z at a resolution of 240,000. MS2 scans utilized 299 isolation windows (2 Th) with a normalized collision energy of 25% HCD. Raw MS data were processed using DIA‐NN (v1.8.1) in a library‐free mode searched against the UniProt mouse reference proteome (UP000000589). The Match Between Runs (MBR) algorithm was implemented to maximize proteome coverage. To ensure high‐confidence identifications, the false discovery rate (FDR) was strictly controlled at < 1% at both the protein and precursor levels.

### 2.8. RNA Stability Assay

To evaluate mRNA stability, HEI‐OC1 cells expressing either WT or mutant *Lmx1a* were seeded into 6‐well plates and cultured overnight to achieve 60%–70% confluency. Cells were subsequently treated with 5 *μ*g/mL Actinomycin D (HY‐17559, MedChemExpress) to block de novo transcription. Total RNA was harvested at predetermined time points (0, 1, 2, 4, and 6 h) posttreatment using TRIzol reagent, with the 0 h group receiving an equivalent volume of dimethyl sulfoxide (DMSO) as a vehicle control. RNA concentration and purity were verified using a NanoDrop spectrophotometer. Subsequently, the extracted RNA was reverse‐transcribed, and the residual *Lmx1a* mRNA levels were quantified by RT‐qPCR. The relative mRNA abundance at each time point was calculated using the 2^−*ΔΔ*Ct^ method, normalized to the internal control *18S rRNA* (primer sequences are provided in Table S3), and calibrated to the 0 h baseline (defined as 100%).

### 2.9. Statistical Analysis

Data are presented as mean ± standard error of the mean (SEM) derived from at least three independent experiments. Statistical analyses and graphical visualizations were executed using GraphPad Prism (Version 9.0, GraphPad Software, San Diego, California, United States) and established Bioconductor packages. Statistical significance between groups was determined using Student′s *t*‐test for two‐group comparisons or a one‐way analysis of variance (ANOVA) followed by an appropriate post hoc test for multiple comparisons. Levels of statistical significance are indicated as follows:  ^∗^ *p* < 0.05,  ^∗∗^ *p* < 0.01,  ^∗∗∗^ *p* < 0.001, and NS indicates not significant (*p* ≥ 0.05).

## 3. Results

### 3.1. Clinical Evaluation

**Figure 2:**
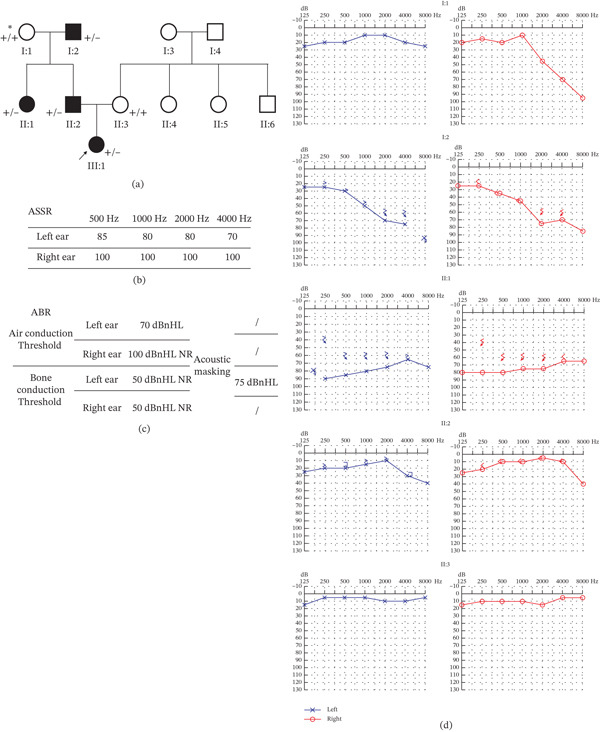
(a) Pedigree with segregation of *LMX1A* c.405delT. Affected status and genotype are indicated: Filled symbols represent the affected individuals. ∗, Nongenetic, sudden sensorineural hearing loss (LMX1A c.405delT negative). (b–c) Audiograms of the proband III: 1 demonstrating severe–profound ASNHL. (d) PTA of I: 1, I: 2, II: 1, II: 2, and II: 3.

**Figure 3:**
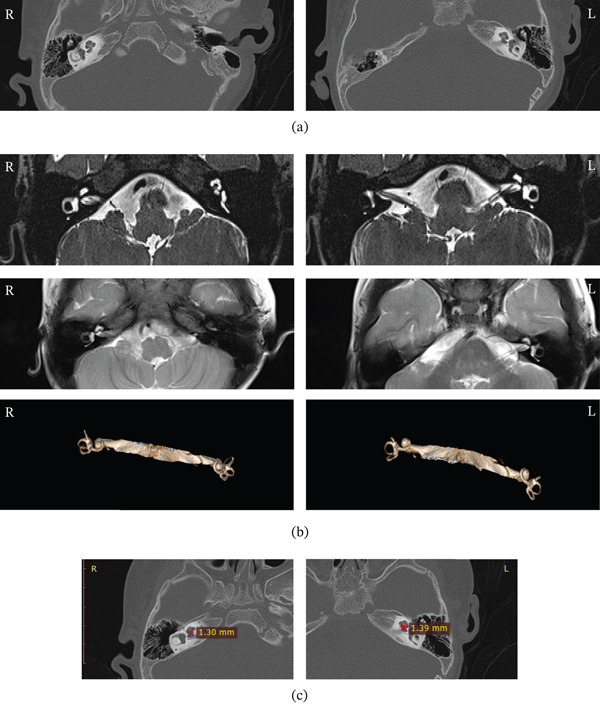
(a–b) Proband III: 1 temporal bone CT/MRI showed inner ear structures. Large vestibular aqueduct was excluded. (c) HRCT revealed cochlear aperture stenosis bilaterally with asymmetric severity (the final cochlear aperture width was calculated based on the average measurements obtained by the two reviewers and defined as cochlear aperture < 1.4 mm).

Audiometric evaluations were performed for six relatives (I: 1, I: 2, II: 1, II: 2, II: 3, and III: 1). Four individuals were diagnosed with NSHL (I: 2, II: 1, II: 2, and III: 1) (Figure 2a). The proband (III: 1), a 3‐year‐old girl, presented with congenital severe‐to‐profound ASNHL. She failed the newborn hearing screening in the right ear and had absent responses on ASSR and ABR bilaterally (Figure 2b,c). HRCT and MRI revealed bilateral CAS (Figure 3a–c). She was fitted with bilateral hearing aids, achieving near‐normal speech perception. Individual II: 1 developed symmetric, bilateral severe SNHL across frequencies in early childhood and uses bilateral hearing aids. Individual II: 2 exhibited high‐frequency SNHL with no documented history of exposure to known ototoxic agents. PTA revealed elevated left‐ear thresholds at 4 and 8 kHz, exceeding the age‐and‐sex matched 95th percentile of normal hearing as defined by the International Organization for Standardization (ISO) 7029: 2017 standard. Individual I: 2 developed progressive, symmetric high‐frequency SNHL in his early 30s. Individual II: 3 presented with normal hearing thresholds. Individual I: 1 experienced unilateral sudden sensorineural hearing loss (SSNHL) in her 40s (Figure 2d). However, she does not carry the familial *LMX1A* variant, suggesting this SSNHL event is an independent phenocopy unrelated to the familial *LMX1A*‐associated HL trait. None of the evaluated individuals exhibited any syndromic features or clinically significant vestibular dysfunction.

### 3.2. Identification and Classification of a Novel Frameshift Variant of *LMX1A*

**Figure 4:**
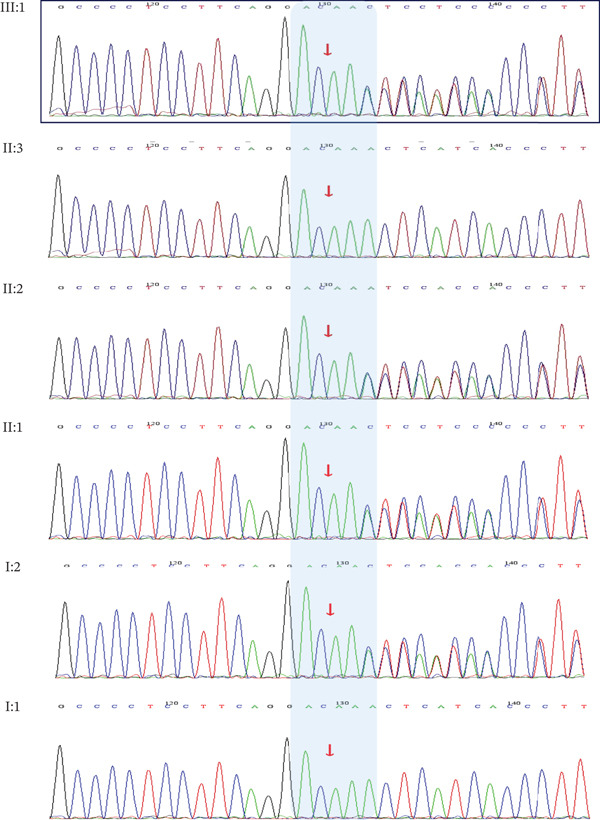
Sanger chromatograms confirming presence and absence of c.405delT in family members. Heterozygous frameshift mutation (red arrows) in affected individuals. Reference sequence: NM_177398.4.

To elucidate the genetic etiology underlying the observed familial HL, WES was performed on the proband (III: 1), her father (II: 2), and her unaffected mother (II: 3). This analysis identified a novel heterozygous frameshift variant in *LMX1A* (c.405delT, p.Phe135LeufsTer3; RefSeq NM_177398.4), shared by the proband and her father. Sanger sequencing confirmed cosegregation of this variant with the auditory phenotype in all available affected family members (I: 2, II: 1, II: 2, III: 1), whereas it was absent from unaffected relatives (I: 1, II: 3) (Figure 4). In addition, we did not identify known pathogenic variants or potential modifier variants in all individuals that could account for the severe phenotypic divergence.

**Figure 5:**
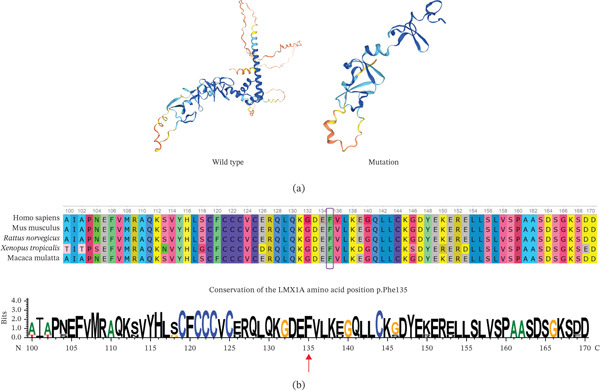
(a) Predicted truncation within LIM2 removing the homeodomain and C‐terminus. (b) Multiple sequence alignment indicating conservation of p.Phe135 across vertebrates.

Located within exon 4, this mutation alters the amino acid at position 135 from phenylalanine (Phe) to leucine (Leu), introducing a premature termination codon (PTC) three residues downstream. This truncation occurs within the LIM2 domain and removes the entire homeodomain and C‐terminus of the protein (Figure 5a). Consequently, the predicted truncated protein would lack the essential structural motifs required for DNA binding and transcriptional regulation. Furthermore, MSA demonstrated that the Phe residue at position 135 is highly conserved among vertebrate LMX1A orthologs (Figure 5b). Based on its cosegregation with the HL phenotype, absence from population databases, and predicted deleterious effect (premature termination), we classified the *LMX1A* c.405delT variant as the causal variant underlying DFNA7 in this family.

### 3.3. Effects of the *LMX1A* Variants on Transcriptional Activity

**Figure 6:**
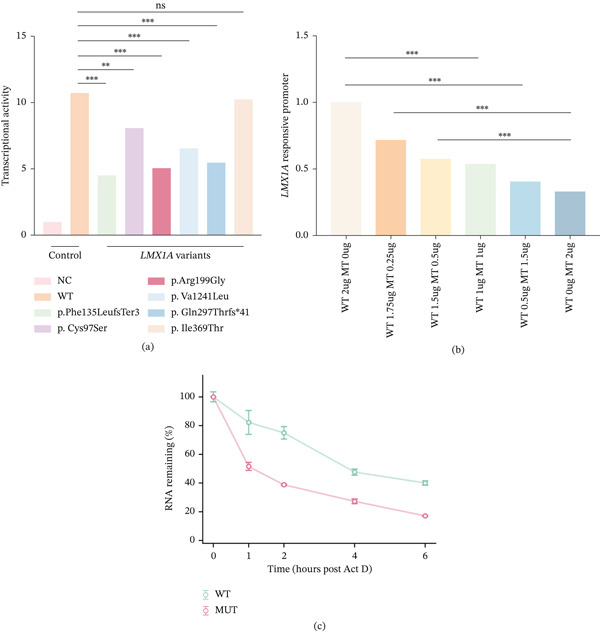
(a) Luciferase reporter assays showing reduced transactivation by LMX1A mutants relative to WT, p.Ile369Thr retains WT‐like activity. Data represent mean ± SEM, statistics by one‐way ANOVA. (b) WT: MT (p.Phe135LeufsTer3) titration demonstrates dose‐dependent reduction without dominant‐negative suppression. Statistical significance: ∗*p* < 0.05, ∗∗*p* < 0.01, ∗∗∗*p* < 0.001, NS (not significant, *p* ≥ 0.05). (c) Accelerated mRNA degradation of the mutant LMX1A transcript. Actinomycin D (ActD) chase assays were performed in transfected HEI‐OC1 cells to evaluate mRNA stability. Cells were treated with 5 *μ*g/mL ActD to block de novo transcription, and remaining *LMX1A* mRNA levels were quantified via RT‐qPCR at the indicated time points (0, 1, 2, 4, and 6 h). The relative mRNA remaining was calculated using the method, normalized to *18S rRNA*, and calibrated to the 0 h baseline. The mutant transcript (red line) degraded noticeably faster than the WT transcript (green line), characteristic of active NMD. Data are presented as mean ± SD.

The p.Phe135LeufsTer3 mutation occurs within the LIM2 domain of *LMX1A*, which is a zinc‐binding motif essential for mediating protein–protein interactions [20]. To functionally evaluate the transactivation capacity of the novel LMX1A p.Phe135LeufsTer3 variant and compare its effect against other domain‐specific variants, we assessed the transcriptional activity of the *INS* promoter in HEK293 cells cotransfected with WT or MT LMX1A expression constructs alongside an *INS*‐luc reporter. Normalized relative luciferase activity (firefly output normalized to the Renilla internal control) was statistically compared between cells expressing each MT LMX1A and the WT construct. Relative to WT LMX1A, all tested dominant mutants (p.Phe135LeufsTer3, p.Cys97Ser, p.Arg199Gly, p.Val241Leu, and p.Gln297ThrfsTer41) exhibited significantly reduced transcriptional activity, whereas the p.Ile369Thr mutant retained transactivation capacity comparable to WT. These findings confirm a profound impairment in the ability of dominant LMX1A mutants to activate downstream target gene transcription (Figure 6a). To distinguish between haploinsufficiency and a dominant‐negative mechanism for the p.Phe135LeufsTer3 variant, we performed plasmid titration experiments cotransfecting WT and MT LMX1A (p.Phe135LeufsTer3) plasmids at various molar ratios (WT: MT from 2: 0 to 0: 2). Reporter activity declined proportionally with increasing mutant plasmid fraction, with dropping below the WT‐only level at any ratio (Figure 6b). This dose‐dependent reduction indicates the absence of a dominant‐negative effect, substantiating haploinsufficiency as the primary functional deficit. Given the early premature stop codon, the mutant transcript is likely subject to nonsense‐mediated decay (NMD), which would further reduce effective LMX1A dosage in vivo.

### 3.4. The *LMX1A* c.405delT Variant Triggers Rapid mRNA Degradation

To determine whether the PTC introduced by the *LMX1A* c.405delT variant affects mRNA stability, we conducted an Actinomycin D chase assay in transfected HEI‐OC1 cells. Following the pharmacological blockade of de novo transcription, total RNA was harvested at 0, 1, 2, 4, and 6 h, and the residual *Lmx1a* transcript abundance was quantified via RT‐qPCR. Time‐course analysis revealed that the mutant *Lmx1a* mRNA degraded at a substantially accelerated rate relative to the WT transcript. By 4 h posttreatment, the relative residual mRNA level of the mutant was lower than that of the WT counterpart (Figure 6c). This accelerated decay profile is characteristic of NMD, a conserved surveillance pathway elicited by PTC‐harboring transcripts.

### 3.5. Integrated Transcriptomic and Proteomic Profiling Reveals Cellular Stress and Inflammatory Signatures

**Figure 7:**
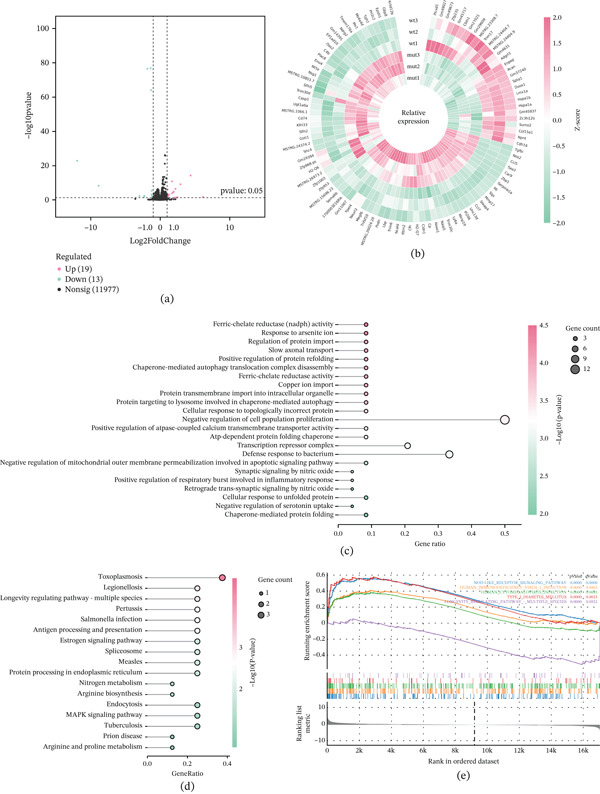
(a) Volcano plot of DEGs. Filter criteria: Benjamini–Hochberg (BH) correction, |log2FC| > 1, and FDR ≤ 0.05. (b) The circular heatmap displays the relative expression patterns (*Z*‐scores) of 100 core functional genes across the biological replicates of mutant (mut1‐3) and wild type (wt1‐3) groups. Genes are clustered based on their expression profiles. (c) GO and (d) KEGG enrichment (FDR ≤ 0.05) showing immune/inflammatory, synaptic transmission/neurodevelopment, proteostasis and ER stress, oxidative stress and metabolic pathways. (e) GSEA enrichment plots display a positive normalized enrichment score (NES) indicating pathway upregulation in the mutant group (NOD‐like receptor signaling pathway and cytosolic DNA‐sensing pathway), whereas a negative NES denotes downregulation (longevity regulating pathway). Statistical significance was determined by the *q*‐value.

Initial transcriptomic profiling of HEI‐OC1 cells expressing the *Lmx1a* c.405delT variant identified significantly differentially expressed genes (DEGs) defined by stringent criteria of |log2FC| > 1 and FDR ≤ 0.05 (Figure 7a). To visualize the expression patterns of the most prominent DEGs, we generated a circular heatmap encompassing 100 core functional genes (Figure 7b). Unsupervised hierarchical clustering strictly segregated the MT and WT groups, with the dysregulated modules prominently featuring sterile inflammation and innate immunity (such as *Zbp1*, *Oas2*, *Il6*), proteostasis and endoplasmic reticulum (ER) stress (*Hspa1a*, *Hspa1b*), and extracellular matrix remodeling (*Mmp17*, *Col15a1*). Consistent with this expression clustering, Gene Ontology (GO) [21, 22] and Kyoto Encyclopedia of Genes and Genomes (KEGG) [23–25] pathway enrichment analyses highlighted focused perturbations in immune‐inflammatory cascades, ER stress, and metabolic homeostasis (Figure 7c,d). Subsequent Gene Set Enrichment Analysis (GSEA) revealed upregulation of immune cascades in the mutant cells. Conversely, networks governing intrinsic cytoprotection and cellular survival were profoundly suppressed. Collectively, these transcriptomic signatures portray a pathological cellular state characterized by exacerbated inflammation and the concurrent collapse of intrinsic resilience mechanisms (Figure 7e).

**Figure 8:**
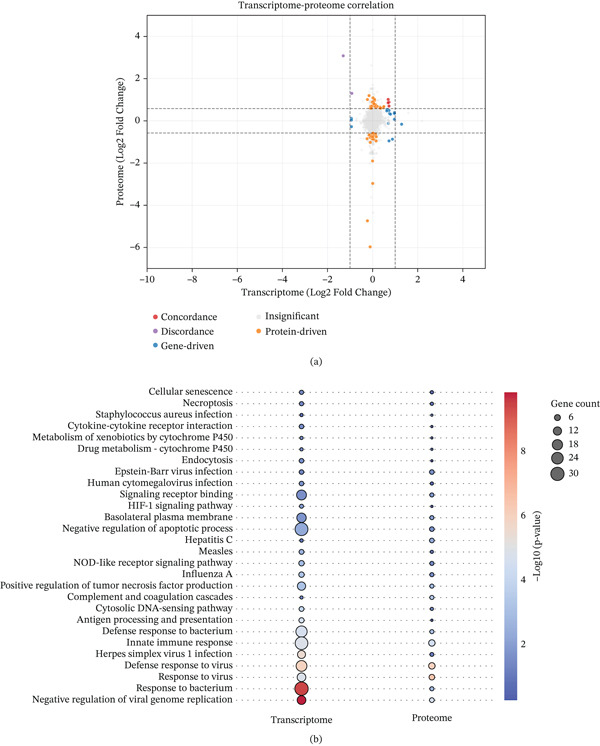
(a) Nine‐quadrant scatter plot of integrated transcriptomic and proteomic data. The analysis illustrates the correlation between mRNA (log2FC) and protein (log2FC) expression profiles in HEI‐OC1 cells expressing the mutant versus WT LMX1A. The dashed lines indicate the significance thresholds |log2FC − gene| > 1|log2FC − prot|0.58. (b) Dual‐omics GO/KEGG bubble plot integrated GO biological processes and KEGG pathways significantly enriched among the differentially expressed molecules are shown. The *y*‐axis represents the enriched terms, whereas the *x*‐axis denotes the GeneRatio.

Building upon the transcriptomic findings an exploratory DIA‐based quantitative proteomic analysis was conducted to elucidate downstream molecular perturbations at the translational level. A nine‐quadrant scatter plot was constructed to globally assess the regulatory correlation between the mRNA and protein strata, this mapping illustrated the distribution of concordantly and discordantly expressed molecules, thereby revealing a complex posttranscriptional regulatory landscape (Figure 8a). To decipher the functional implications of these multiomics alterations, a dual‐modality enrichment analysis was performed to isolate biological pathways cross‐validated at both the RNA and protein levels. This dual‐validation approach revealed synchronized enrichment in proteostasis and ER stress networks (Figure 8b), specifically highlighting terms such as cellular response to topologically incorrect protein, chaperone‐mediated autophagy, and protein processing in ER. Immune and inflammatory cascades were robustly activated. Despite the sterile in vitro environment, pathways associated with the innate immune response, inflammatory respiratory burst, and infectious disease proxies were highly enriched. This inflammatory signature was accompanied by synchronized upregulation trends in Zbp1 and NOD‐like receptor signaling components across both omics layers. Furthermore, the integrated analysis uncovered perturbations in metabolic and ionic homeostasis, evidenced by the significant enrichment of arginine biosynthesis, calcium transmembrane transporter activity, and mitochondrial outer membrane permeabilization. Synaptic and neural signaling pathways, including slow axonal transport and asymmetric synapse organization, were similarly dysregulated. Concurrently, the omics data indicated a depletion of cytoprotective networks and key cytoskeletal proteins, culminating in the enrichment of the necroptosis pathway.

### 3.6. Pathogenicity Classification According to ACMG/AMP Guidelines

According to the established standards and guidelines of the American College of Medical Genetics and Genomics/Association for Molecular Pathology (ACMG/AMP) [26] and applying the specific recommendations for evaluating loss‐of‐function variants [27], the novel *LMX1A* variant c.405delT (p.Phe135LeufsTer3) was classified as pathogenic. The primary line of evidence is PVS1 (very strong); this frameshift variant introduces a PTC within the LIM2 domain, completely truncating the critical DNA‐binding homeodomain and C‐terminus. Loss of function is a well‐established disease mechanism for DFNA7, and the variant occurs upstream of previously reported pathogenic nonsense variants [28]. Importantly, this genetic finding is corroborated by our in vitro functional validation, which demonstrated significantly impaired transcriptional activity, thereby fulfilling the criteria for PS3 (strong). Furthermore, the variant is absent from large population cohorts, including the gnomAD, providing evidence for PM2 (moderate). Additionally, its cosegregation with the HL phenotype across multiple affected individuals in the family supports PP1 (supporting). Collectively, the combination of PVS1, PS3, PM2, and PP1 provides robust evidence for the pathogenicity of this novel variant.

## 4. Discussion

In this study, we identify a novel frameshift variant in *LMX1A* (c.405delT, p.Phe135LeufsTer3) that cosegregates with DFNA7 in a three‐generation family, thereby broadening the *LMX1A* mutational spectrum. Affected individuals exhibited pronounced phenotypic variability, characterized by HL severity ranging from mild to profound, symmetric or asymmetric presentations, and onset ages spanning from congenital to adulthood.

*LMX1A* is located on chromosome 1q32 and encodes a conserved LIM domain transcription factor that is essential for the development of both the auditory system and various neuronal populations. Structurally, the LMX1A protein comprises two N‐terminal zinc‐binding LIM domains (LIM1 and LIM2) that mediate protein–protein interactions, alongside a C‐terminal DNA‐binding homeodomain. This architecture enables LMX1A to function as a scaffold for the assembly of tissue‐specific transcriptional complexes. Beyond its well‐documented role in orchestrating cell fate decisions [29, 30] and neuronal differentiation within the central nervous system, particularly for midbrain dopaminergic neurons [29, 31–35], LMX1A acts as a crucial upstream regulator of inner ear morphogenesis, ensuring the proper development of the cochlear and vestibular apparatus [36–39]. Its activity is further refined by negative regulators such as *LMO4*, which prevent ectopic sensory formation within the cochlea [40]. Although the predominant human phenotype involves inner ear dysfunction leading to HL, animal models have revealed broader developmental anomalies affecting cochlear structure [38, 39].

To date, the majority of reported pathogenic *LMX1A* variants have been mapped to the homeodomain or the C‐terminal region. In this study, we identified and functionally validated the pathogenicity of a novel variant situated in the LIM2 domain of LMX1A, affecting a highly conserved residue. Depending on their location, disease‐associated variants disrupt LMX1A function via distinct mechanisms; homeodomain mutations typically impair DNA binding, whereas LIM domain variants likely disrupt protein–protein interactions and cofactor recruitment. This mechanistic heterogeneity underscores the pleiotropic developmental role of LMX1A and partially explains its association with diverse clinical phenotypes.

Mechanistically, haploinsufficiency emerges as the primary pathogenic mechanism of *LMX1A*‐associated ADNSHL [9, 41]. Although a single functional *LMX1A* allele is sufficient for overall embryonic development, it is inadequate to maintain normal cochlear and vestibular function. Consequently, heterozygous loss‐of‐function variants lead to critically reduced LMX1A dosage in specific cochlear cell types, ultimately manifesting as progressive HL with variable severity and expressivity. Consistent with this mechanism, our functional assays demonstrated a profound loss of transactivation capacity for multiple *LMX1A* variants affecting the LIM domains and homeodomains (p.Phe135LeufsTer3, p.Cys97Ser, p.Arg199Gly, p.Val241Leu, and p.Gln297ThrfsTer41), whereas the recessive p.Ile369Thr variant retained wild‐type‐like activity. Importantly, the implementation of plasmid titration assays to model allelic imbalance (varying WT: MT ratios) for the p.Phe135LeufsTer3 variant explicitly provides solid functional evidence for haploinsufficiency. This rigorous validation strengthens the genotype‐phenotype correlation and establishes a firm molecular basis for our clinical observations. This haploinsufficiency model is further corroborated by the Actinomycin D chase assay, which revealed that the mutant mRNA degrades at a noticeably accelerated rate relative to the wild‐type transcript, validating the activation of NMD.

Multiomics profiling revealed significant disruptions across cellular homeostasis and stress pathways, suggesting a complex cellular stress response induced by *LMX1A* haploinsufficiency. Integrated GO and KEGG pathway analyses identified a significant enrichment of terms associated with ER stress, oxidative stress, and synaptic transmission. Furthermore, GSEA demonstrated an upregulation of immune and inflammatory‐related cascades, such as the NOD‐like receptor and cytosolic DNA‐sensing signaling pathways, alongside a downregulation of the longevity regulating pathway. Our findings from the Actinomycin D assay confirmed that the *Lmx1a* c.405delT transcript undergoes NMD, supporting haploinsufficiency as the primary mechanism. It is plausible that the perturbations in proteostasis and ER stress observed in the multiomics analysis represent a generalized cellular stress response secondary to the disruption of the *Lmx1a*‐dependent regulatory network. However, we cannot preclude the possibility that these alterations partially reflect an intrinsic, nonspecific response of the artificial HEI‐OC1 overexpression system to in vitro manipulation. Therefore, the precise mechanisms driving this haploinsufficiency‐induced stress response remain to be verified by further investigation.

Given that immune factors are widely expressed in the human cochlea, specifically in the organ of Corti, as well as the spiral ganglion and spiral ligament [42–46], we propose that this resulting sterile inflammation may contribute significantly to auditory pathogenesis, paralleling recent studies linking *LMX1A* dysfunction to neuroinflammation and neurodegeneration [47, 48]. Concurrently, the loss of proper LMX1A transcriptional regulation likely perturbs vital ionic networks and dysregulates synaptic vesicle release, depriving auditory cells of intrinsic resilience [49–51], and rendering them acutely vulnerable to excitotoxicity. Collectively, prolonged ER stress, ionic imbalance, and chronic sterile inflammation could eventually exhaust homeostatic reserves, potentially leading to hair cell cytoskeletal degradation. This time‐dependent cumulative toxicity parallels the progressive nature of the observed HL. Additionally, the stochastic nature of local cellular homeostatic reserve depletion could hypothetically account for its asymmetric onset. We acknowledge that these proposed downstream cascades are currently inferred from omics signatures and require future validation.

Developmentally, LMX1A exhibits a highly dynamic and biphasic expression pattern during inner ear organogenesis. Early in embryogenesis, it is broadly expressed throughout the otic epithelium, acting as a pioneering transcription factor essential for dorsal‐ventral axis establishment and the segregation of neurogenic, sensory, and nonsensory domains [52, 53]. As inner ear morphogenesis progresses, the expression domain of LMX1A undergoes profound spatial restriction. During later developmental and maturation stages, LMX1A expression becomes compartmentalized exclusively within nonsensory epithelia, such as the endolymphatic duct, the roof of the cochlear duct, and the greater epithelial ridge, whereas it is downregulated or virtually absent in mature sensory hair cells [36, 52]. This highly compartmentalized expression provides a developmental framework for the observed clinical phenotypes. Specifically, the disruption of *LMX1A-*mediated regionalization of the otic capsule and membranous labyrinth during early development, driven by haploinsufficiency, provides a theoretical developmental rationale for structural anomalies such as CAS observed in the proband. Furthermore, although mature hair cells are not the primary expressing cell type for LMX1A, the adjacent nonsensory epithelia are critical for maintaining endolymphatic homeostasis and the endocochlear potential. Cellular stress within these compartments could precipitate a secondary, chronic microenvironmental deterioration. This disruption gradually compromises hair cell survival, further explaining the progressive nature of the SNHL.

Clinically, congenital ASNHL is a complex trait frequently linked to cochlear nerve deficiency [54, 55], acquired ototoxic or viral insults [56], and genetic variants in *GJB2* [57, 58] and *SLC26A4* [59]. Against this backdrop, the specific mechanisms underlying *LMX1A*‐related ASNHL warrant consideration. Although the exact mechanisms remain elusive, previous studies suggest that incomplete penetrance or impaired lateralization pathways might play a role [5]. Diminished penetrance in one ear could predispose it to more severe degeneration of inner ear hair cells, ultimately exacerbating the auditory phenotype. Concepcion et al. suggested that regulating *LMX1A* downstream targets can rescue laterality defects underlying ASNHL and modulate compensatory mechanisms that influence HL severity [60]. Notably, the proband exhibited ASNHL concurrent with bilateral CAS on imaging, suggesting a potential structural basis for this clinical asymmetry [61].

Beyond these structural correlates, pathogenic variants of *LMX1A* are associated with both AD and AR forms of NSHL. Although the majority of cases follow an AD inheritance pattern linked to the DFNA7 locus, a homozygous missense variant (c.1106 T > C, p.Ile369Thr) in the C‐terminus has been reported to cause ARNSHL in a single Pakistani family [7], highlighting the allelic heterogeneity of *LMX1A*‐related HL. Clinically, the DFNA7 phenotype is characterized by marked variable expressivity, encompassing wide differences in onset age, severity, symmetry, and the presence of vestibular dysfunction; our findings align with and further expand upon this clinical spectrum. We observed intrafamilial variable expressivity, with the proband presenting with profound HL and CAS, in contrast to her father′s milder, high‐frequency phenotype. Furthermore, the absence of previously reported manifestations, such as endolymphatic hydrops [62] in this family underscores this high degree of phenotypic variability. Several limitations of the present study should be acknowledged. Given the WES was performed, we explicitly screened for potential modifier variants in other known HL‐genes; no significant pathogenic variants or modifier genes were identified. It is important to note that WES is inherently limited in its ability to detect noncoding regulatory variants and to systematically assess complex modifier loci. Consequently, whether the intrafamilial clinical divergence is driven by deep intronic variants undetected by WES, epigenetic regulations, or specific environmental factors remains unresolved. Additionally, the lack of longitudinal audiometric data limits our ability to determine whether the asymmetry phenotype observed in childhood becomes more symmetric over time, and confounding factors such as age‐related HL in older family members cannot be entirely excluded. Finally, the unavailability of temporal bone imaging for other affected relatives precludes a comprehensive assessment of CAS prevalence across the pedigree. Collectively, these limitations highlight the constraints of our current single‐pedigree analysis and underscore the necessity for comprehensive modifier screening and longitudinal tracking in future large‐cohort studies to refine these genotype‐phenotype correlations.

Importantly, the transcriptomic and proteomic findings presented here remain exploratory as they derived from an in vitro cell model. To bridge this gap and fully elucidate the precise spatio‐temporal pathogenic cascade of the *LMX1A* c.405delT variant, generating in vivo animal models and establishing patient‐derived induced pluripotent stem cells (iPSCs) represent critical next steps.

## 5. Conclusion

In line with the above, we have identified the *LMX1A* (c.405delT, p.Phe135LeufsTer3) variant as the genetic cause of progressive and asymmetric SNHL in this pedigree. The associated clinical phenotype exhibits pronounced intrafamilial variable expressivity, and we note the presence of bilateral CAS as an associated radiologic finding in the proband. Biochemical assays, including plasmid titration and expression monitoring, functionally corroborated haploinsufficiency as the principal disease mechanism. Furthermore, exploratory multiomics profiling reveals signatures consistent with perturbation in auditory cellular homeostasis characterized by disrupted synaptic signaling and exacerbated sterile inflammation. This study provides critical genomic evidence to enhance genetic counseling and offers robust mechanistic insights to guide future studies and potential therapeutic strategies for *LMX1A*‐related HL.

## Author Contributions

Chenyang Xu, Suyang Wang, and Xiaowen Liu provided the diagnosis, examination, and treatment of the patients. Chenyang Xu and Zhipeng Nie wrote the article. Chenyang Xu, Suyang Wang and Yiming Zhu analyzed the data. Yufen Guo reviewed all content and gave guidance. Yufen Guo and Xiaowen Liu contributed to the work equally and should be regarded as co‐corresponding authors. Chenyang Xu, Zhipeng Nie, and Suyang Wang contributed to the work equally and should be regarded as co‐first authors.

## Funding

This study was supported by National Natural Science Foundation of China (10.13039/501100001809) (Nos. 81960192, 82160214); Natural Science Foundation of Gansu Province (10.13039/501100004775), 25JRRA340); and Maternal and Child Health Hospital of Gansu Province (RCBS‐2023‐001).

## Ethics Statement

This study was approved by the Committee of Medical Ethics of Lanzhou University Second Hospital.

## Consent

All authors consented to any form of data contained in this study.

## Conflicts of Interest

The authors declare no conflicts of interest.

## Supporting information


**Supporting Information** Additional supporting information can be found online in the Supporting Information section. **Supporting Information.** Table S1: Primer information for PCR amplification of variant loci of the LMX1A gene. Table S2: −613 bp upstream INS promoter region‐pGL4.12. Table S3: Primer information for real‐time PCR amplification.

## Data Availability

The data that support the findings of this study are openly available in GEO at Https://Www.Ncbi.Nlm.Nih.Gov/Geo/, Reference Number GSE299979.
