# Supplementary material for: A Novel LMX1A Frameshift Variant Underlies Familial Phenotypic Heterogeneity in DFNA7
Source: Hum Mutat. 2026 May 30;2026:9930672. doi: 10.1155/humu/9930672 (PMC13240465; doi:10.1155/humu/9930672)
Supplement: Supplementary file 1 — Supporting Information Additional supporting information can be found online in the Supporting Information section. Supporting Information. Table S1: Primer information for PCR amplification of variant loci of the LMX1A gene. Table S2: −613 bp upstream INS promoter region‐pGL4.12. Table S3: Primer information for real‐time PCR amplification. [file HUMU-2026-9930672-s001.docx]

| **Subject** | **ds SNP** | **Genotype** | **Forward primer（5'→3'）** | **Reverse primer（5'→3'）** |
| --- | --- | --- | --- | --- |
| **Ⅲ:1** | rs1652976134 | *LMX1A*/het | AAACAATGCAGACAGCCACC | TCATATCCCAGTTCTTGAAGTTCC |
| **Ⅱ:2** | rs1652976134 | *LMX1A*/het | AAACAATGCAGACAGCCACC | TCATATCCCAGTTCTTGAAGTTCC |
| **Ⅱ:3** | rs1652976134 | Wild type | AAACAATGCAGACAGCCACC | TCATATCCCAGTTCTTGAAGTTCC |
| **Ⅱ:1** | rs1652976134 | *LMX1A*/het | AAACAATGCAGACAGCCACC | TCATATCCCAGTTCTTGAAGTTCC |
| **Ⅰ:2** | rs1652976134 | *LMX1A*/het | AAACAATGCAGACAGCCACC | TCATATCCCAGTTCTTGAAGTTCC |
| **Ⅰ:1** | rs1652976134 | Wild type | AAACAATGCAGACAGCCACC | TCATATCCCAGTTCTTGAAGTTCC |

**Supplementary Table1:** Primer information for PCR amplification of the variant loci of the *LMX1A* gene.


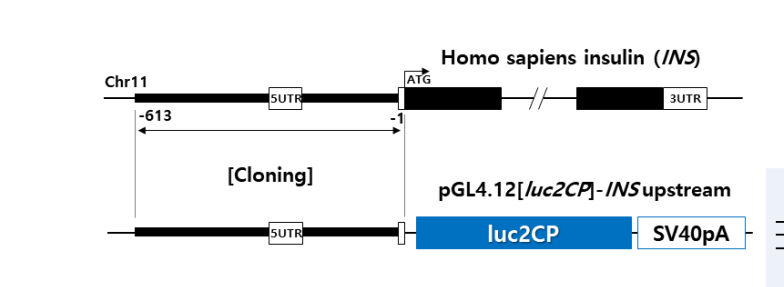


ggggtctggggacagcagcgcaaagagccccgccctgcagcctccagctctcctggtctaatgtggaaagtggcccaggtgagggctttgctctcctggagacatttgcccccagctgtgagcagggacaggtctggccaccgggcccctggttaagactctaatgacccgctggtcctgaggaagaggtgctgacgaccaaggagatcttcccacagacccagcaccagggaaatggtccggaaattgcagcctcagcccccagccatctgccgacccccccaccccaggccctaatgggccaggcggcaggggttgagaggtaggggagatgggctctgagactataaagccagcgggggcccagcagccctcagccctccaggacaggctgcatcagaagaggccatcaagcaggtctgttccaagggcctttgcgtcaggtgggctcaggattccagggtggctggaccccaggccccagctctgcagcagggaggacgtggctgggctcgtgaagcatgtgggggtgagcccaggggccccaaggcagggcacctggccttcagcctgcctcagccctgcctgtctcccagatcactgtccttctgcc

**Supplementary table 2:** -613bp upstream INS promoter region-pGL4.12

| **Gene** | **Forward primer（5'→3'）** | **Reverse primer（5'→3'）** |
| --- | --- | --- |
| ***LMX1A*** | CAGGGAAAGGAACTGCTGAG | CTGGTTTTGGAACCACACCT |
| ***GADPH*** | AACGGATTTGGTCGTATTGGG | CCTGGAAGATGGTGATGGGAT |
| ***18S rRNA*** | GATGGTAGTCGCCGTGCC | CCAAGGAAGGCAGCAGGC |

**Supplementary Table 3:** Primer information for Real-Time PCR amplification.
